# Apelin alleviated neuroinflammation and promoted endogenous neural stem cell proliferation and differentiation after spinal cord injury in rats

**DOI:** 10.1186/s12974-022-02518-7

**Published:** 2022-06-20

**Authors:** Qing Liu, Shuai Zhou, Xiao Wang, Chengxu Gu, Qixuan Guo, Xikai Li, Chunlei Zhang, Naili Zhang, Luping Zhang, Fei Huang

**Affiliations:** 1 Institute of Neurobiology, Binzhou Medical University, 346 Guanhai Road, Laishan, 264003 Shandong China; 2 School of Health and Life Sciences, University of Health and Rehabilitation Sciences, 17 Shandong Road, Qingdao, 266071 China

**Keywords:** Apelin, SCI, Neuroinflammation, Endogenous neural stem cells, Astrocytes

## Abstract

**Background:**

Spinal cord injury (SCI) causes devastating neurological damage, including secondary injuries dominated by neuroinflammation. The role of Apelin, an endogenous ligand that binds the G protein-coupled receptor angiotensin-like receptor 1, in SCI remains unclear. Thus, our aim was to investigate the effects of Apelin in inflammatory responses and activation of endogenous neural stem cells (NSCs) after SCI.

**Methods:**

Apelin expression was detected in normal and injured rats, and roles of Apelin in primary NSCs were examined. In addition, we used induced pluripotent stem cells (iPSCs) as a carrier to prolong the effective duration of Apelin and evaluate its effects in a rat model of SCI.

**Results:**

Co-immunofluorescence staining suggested that Apelin was expressed in both astrocytes, neurons and microglia. Following SCI, Apelin expression decreased from 1 to 14 d and re-upregulated at 28 d. In vitro, Apelin promoted NSCs proliferation and differentiation into neurons. In vivo, lentiviral-transfected iPSCs were used as a carrier to prolong the effective duration of Apelin. Transplantation of transfected iPSCs in situ immediately after SCI reduced polarization of M1 microglia and A1 astrocytes, facilitated recovery of motor function, and promoted the proliferation and differentiation of endogenous NSCs in rats.

**Conclusion:**

Apelin alleviated neuroinflammation and promoted the proliferation and differentiation of endogenous NSCs after SCI, suggesting that it might be a promising target for treatment of SCI.

## Introduction

Spinal cord injury (SCI) is a devastating condition affecting millions of people worldwide annually [1]. The prognosis of SCI is closely related to secondary injury [2], a process dominated by neuroinflammation. Therefore, inhibiting inflammasome activation, reducing pro-inflammatory cytokine production, and improving the local spinal cord microenvironment are promising therapeutic strategies for SCI [3, 4].

Spinal cord injury can prime microglia towards M1 microglia phenotype, which is responsible for provoking neuroinflammation [5], meanwhile, M1 microglia induces activation of astrocytes. Reactive astrocytes form a glial scar that obstructs neuronal axonal regeneration and communication [6]. Astrocytes can reportedly be divided into two types: A1 and A2 [7, 8]. A1 astrocytes release inflammatory cytokines that can inhibit proliferation and differentiation of oligodendrocyte precursor cells, and kill nearby neurons. Thus, it is concluded that inhibiting A1-type reactive astrocytes is an important strategy to suppress inflammation and promote repair of nerve injury [9].

Apelin (gene name: Apln), an endogenous ligand that binds the G protein-coupled receptor angiotensin-like receptor 1 (APJ) [10], exhibits a therapeutic effect on central nervous system (CNS) disorders such as stroke, Alzheimer’s disease, and Moyamoya disease [11–13]. A recent study reported that Apelin-13 can promote recovery of rat spinal cord ischemia/reperfusion injury by reducing autophagy [14]. However, preliminary studies evaluating the effect of Apelin on recovery from SCI showed that although it was potentially effective, the short half-life of Apelin peptides greatly limits it therapeutic utility for clinic applications [15, 16]. Therefore, it is important to prolong the duration of action of Apelin.

Our previous study found that neural stem cells (NSCs) enhance nerve regeneration after sciatic nerve injury in rats [17]. However, the toxic microenvironment of the injury is unfavorable for survival of transplanted cells [18]. Previous studies reported that endogenous NSCs were activated after SCI in the central canal of the spinal cord, whereby they took part in the regeneration of neural function [19, 20]. Therefore, promoting the proliferation and differentiation of NSCs can be an effective strategy for treatment of nerve injuries [21].

This study aimed to examine the potential role of Apelin in treatment of SCI. First, we evaluated changes of Apelin expression after SCI to determine whether Apelin was involved in injury or repair processes. Furthermore, to detect the effect of Apelin on NSCs, we assessed in vitro proliferation and differentiation of NSCs after administration of Apelin or its inhibitor ML221. Finally, to address the short half-life of Apelin that greatly limits its therapeutic utility [22], we infected induced pluripotent stem cells (iPSCs) with lentivirus bearing an Apelin expression vector, and then transplanted these cells into the injury in situ to prolong the effective duration of Apelin.

Our findings demonstrate that Apelin expression was decreased after SCI from 1 to 14 days post-injury (dpi), and subsequently upregulated at 28 dpi. Therefore, we hypothesized that Apelin may play a role in cellular and molecular inflammatory cascades of the spinal cord. Our results suggest that Apelin contributes to NSC proliferation and differentiation. Furthermore, transplantation of iPSCs overexpressing Apelin alleviated inflammation associated with secondary damage; enhanced the recovery of motor functions; reduced M1 microglia and A1 astrocyte activation at 14 dpi; and promoted the activation, proliferation, and differentiation of endogenous NSCs in vivo.

In conclusion, our results demonstrate that Apelin may be a novel molecular target for SCI recovery.

## Materials and methods

### Animals

A total of 127 specific pathogen-free 8-week female Sprague-Dawley rats weighing 220–250 g (Jinan PengYue Laboratory Animal Breeding, Jinan, China) were used in experiments. All rats were housed in a separate environment under a 12-h light–dark cycle at 24 ± 2 °C, with 50% relative humidity. Food and water were available ad libitum. Experimental protocols for animals in this study were approved by the Animal Care and Use Committee of Binzhou Medical University. All animals were acclimatized to the new environment for at least 7 days before experiments.

Animal experiments were divided into two parts. Sprague-Dawley rats for Part 1 were randomized into three groups: control (*n* = 6), sham control (*n* = 6), and SCI (*n* = 30). Rats in the SCI group were killed at 1, 3, 7, 14, and 28 dpi. Rats in Part 2 were randomized into five groups: (1) Sham: rats subjected to laminectomy only; (2) SCI: after laminectomy, a transection was made between T9 and T10 with a sharp blade, and 4 µL of iPSC culture medium were locally injected immediately after SCI; (3) H-Apln: 1 × 10^5^ H-Apln iPSCs in 4 µL iPSC culture medium (*Apln*-overexpressing iPSCs) were locally injected immediately after SCI; (4) green fluorescent protein (GFP): 1 × 10^5^ GFP + iPSCs (iPSCs infected with a GFP vector only) in 4 µL iPSC culture medium were locally injected immediately after SCI; and (5) ML221: ML221 (Apelin inhibitor, 30 µg/rat) was intraperitoneally administered 30 min after SCI.

### SCI surgery, cell transplantation, and inhibitor injection

The SCI transection model was established as previously reported [23]. Briefly, rats were anesthetized with 4% chloral hydrate (100 mg/kg body weight; Tianjin DaMao Chemical Reagent Factory, Tianjin, China). The spinal cords of sham group rats were exposed at T8–T10 by laminectomy of these vertebrae. For the SCI transection model, the spinal cord was transected at T9 using a No.11 blade, and the bleeding was controlled using gauze. SCI group rats were immediately locally injected with 4 µL of iPSC culture medium using a microsyringe, while H-Apln group and GFP group rats were given the same volume of culture medium containing 5 × 10^5^ iPSCs (Apln-overexpressing or GFP-infected only, respectively). After surgery, urination was aided twice a day until recovery of the micturition reflex.

### Tissue processing

At designated time points post-injury, rats were anesthetized with 4% chloral hydrate and intracardially perfused with at least 200 mL of 0.9% physiological saline, followed by 400 mL of paraformaldehyde (PFA). Subsequently, 1-cm segments were obtained from the injured lesion.

### Paraffin section histopathological staining

At designated time points after injury, the tissue around the injury site at T8–T10 (about 1 cm) was obtained and fixed in PFA for at least 48 h. Subsequently, the tissue was dehydrated in xylene followed by a gradient series of alcohol, embedded in paraffin, cut into 4-µm serial sections, heated at 60 °C for at least 2 h, and then stored at room temperature.

### Hematoxylin and eosin (HE) staining

Paraffin sections were placed in xylene followed by a gradient series of alcohol, and then stained with hematoxylin for 5 min at room temperature. After rinsing sections in running water, they were differentiated in 1% hydrochloric acid and double-stained with eosin for 3 min. Finally, slides were dehydrated with a gradient of ethanol, permeabilized with xylene, and sealed.

### Luxol Fast Blue (LFB) staining

Paraffin sections were placed in xylene, followed by 100% and 95% ethanol solutions. Next, sections were stained with LFB solution and heated at 60 °C overnight. The next day, sections were washed in 95% ethanol and then placed in 0.05% lithium carbonate aqueous solution for 10 s, followed by 75% alcohol for 30 s; the last two steps were repeated until the white and grey matter were clearly observable. Finally, slides were sealed after dehydration in ethanol and xylene.

### Nissl staining

Paraffin sections were dewaxed and rehydrated before staining with cresyl violet. Subsequently, sections were differentiated in 75% ethanol solution and distilled water until Nissl bodies were clearly observable under a microscope. Numbers of Nissl bodies in the anterior horn were used to detect neuronal damage.

### Serum preparation

5 mL blood samples were taken from the heart tip of rats from normal or injured rats at 1, 3, 7, 14, 28 d post-spinal cord injury, and serum was obtained by centrifuging the blood at 3000 rpm for 10 min at 4 °C.

### Frozen section preparation

Spinal cord tissues around the injured lesion were placed into PFA overnight at 4 °C, dehydrated with sucrose solution (15% for 1 d and 30% for 2 d), embedded in optimum cutting temperature compound, and cut into 12-µm frozen sections with a microtome.

### Immunofluorescence

Frozen sections were equilibrated to room temperature (RT), washed three times (15 min each) with phosphate-buffered saline (pH 7.2–7.4), blocked for 1 h with normal goat serum at RT, and incubated with primary antibody at 4 °C overnight. Following overnight incubation, sections were washed three times and incubated with secondary antibody for 2 h at RT. Finally, sections were washed another three times, nuclear stained with 4,6-diamidino-2-phenylindole (DAPI) for 8 min at RT, and sealed with an anti-fluorescence quencher. Images were obtained under a fluorescent microscope.

The following primary antibodies were used for immunofluorescence at the indicated dilutions: anti-Apelin (1:400; Affinity Biosciences, Zhenjiang, China), anti-glial fibrillary acidic protein (GFAP; ab7260, 1:500; Abcam, Cambridge, UK), anti-GFAP (ab4674, 1:1000, Abcam), anti-Iba1 (1:200, Abcam), anti-Olig2 (1:200; R&D Systems, Minneapolis, MN, USA), anti-NeuN (1:400; Proteintech, Rosemont, IL, USA), anti-Nestin (1:300, Proteintech), anti-C3 (1:200, Proteintech), anti-CD68 (1:200, Abbkine, Wuhan, China), anti-BrdU (1:300, Abbkine), anti-TMEM119 (1:200, Proteintech), anti-iNOS (1:200, Proteintech), anti-Arginase1 (1:300, Proteintech), anti-NF200 (1:200, Proteintech), anti-NeuN (1:50; Cell Signaling Technology, Danvers, MA, USA).

The following fluorescent secondary antibodies were used at a suitable concentration: Alexa Fluor 488 goat-anti-rabbit (Invitrogen, Carlsbad, CA, USA), Alexa Fluor 594 goat-anti-mouse (Abbkine), Alexa Fluor 555 goat-anti-chicken (Bioss, Beijing, China), Alexa Fluor 594 rabbit-anti-goat (Abbkine), and Alexa Fluor 350 goat-anti-rabbit (Abbkine).

### Western blotting

Total protein was extracted from spinal cord tissue using radioimmunoprecipitation assay buffer containing 1% phenylmethanesulfonyl fluoride. Protein concentrations were measured using a BCA assay kit (Beyotime, Shanghai, China). Proteins were resolved by sodium dodecyl sulfate polyacrylamide gel electrophoresis (using gels of different concentrations) and subsequently transferred onto polyvinylidene difluoride membranes. Next, membranes were incubated with the following primary antibodies at 4 °C overnight: anti-Apelin (1:1000; Affinity Biosciences, Zhenjiang, China), anti-Nestin (1:2000, Proteintech), anti-GFAP (1:4000, Abcam), anti-C3 (1:1000, Proteintech), as well as anti-GAPDH (1:10,000, Proteintech) as an internal control. The following day, membranes were washed three times with Tris-buffered saline containing Tween (TBST, 15 min each), incubated with secondary antibodies at room temperature for 2 h, and then washed three times with TBST. Finally, protein bands were visualized using a Bio-Rad Image Lab system (Hercules, CA, USA), and densitometry analysis was performed with ImageJ software (http://imagej.nih.gov).

### Quantitative reverse transcription PCR (qRT-PCR)

Total RNA was extracted from spinal cord tissue with TRIzol (Takara, Kusatsu, Japan) according to the manufacturer’s protocol. To assess the purity and concentration of total RNA templates, 260/280 and 260/230 absorbance ratios were determined using an ultraviolet–visible light spectrophotometer (NanoDrop 2000; Thermo Fisher Scientific, Waltham, MA, USA). A Transcriptor First Strand cDNA Synthesis Kit (Roche, Basel, Switzerland) was used to synthesize cDNA from total RNA (1000 ng/sample). qRT-PCR was carried out using Premix Ex Taq™ (Probe qPCR) (Roche) with GAPDH as an internal reference gene. All primers (refer Table 1 for detailed primer information) were purchased from Accurate Biotechnology (Hunan, China). Data were analyzed by the 2^−ΔΔCT^ method.

**Table 1:** Gene primers tested in comparative RT-PCR experiments

Gene	Forward primer (5′–3′)	Reverse primer (5′–3′)
Apln	CGATGGGAATGGGCTGGAAGA	CAGAAAGGCATGGGTCCCTTATG
Beta-III-tubulin	CAGATGCTGGCCATTCAGAGTAAG	TGTTGCCGATGAAGGTGGAC
GFAP	GCCACCTCAAGAGGAACATCG	CTTGTGCTCCTGCTTCGACTC
IL-1 beta	CCCTGAACTCAACTGTGAAATAGCA	CCCAAGTCAAGGGCTTGGAA
IL-4	TGCACCGAGATGTTTGTACCAGA	TTGCGAAGCACCCTGGAAG
IL-10	CAGACCCACATGCTCCGAGA	CAAGGCTTGGCAACCCAAGTA
TNF-alpha	TCAGTTCCATGGCCCAGAC	GTTGTCTTTGAGATCCATGCCATT
GAPDH	GCACCGTCAAGGCTGAGAAC	TGGTGAAGACGCCAGTGGA

### Enzyme-linked immunosorbent assay (ELISA)

Spinal cord tissues were homogenized in phosphate-buffered saline, followed by centrifugation at 5000×*g* for 10 min to extract protein samples. Protein concentrations in each sample were measured with a BCA assay kit (Beyotime) and adjusted to the same concentration. Apelin expression was measured in serum, while interleukin 1 beta (IL-1β), IL-10, and tumor necrosis factor alpha (TNF-α) were measured in tissue with ELISA kits (Could-Clone Crop, Wuhan, China) according to the manufacturer’s protocols. After detecting the absorbance at a 450 nm wavelength, the concentration of each factor was calculated based on a standard curve.

### iPSC culture and transfection

Human iPSCs (Saibaikang Biotechnology, Shanghai, China) were cultured in mTeSR1 medium. iPSCs were transfected with lentivirus stably expressing Apln or GFP only (Jiman Gene, Shanghai, China). Stably transfected cells were selected by puromycin (MedChemExpress, Monmouth Junction, NJ, USA) before use.

### NSC cultures from newborn rats

Primary spinal cord NSC cultures were prepared from newborn Sprague-Dawley rats (< 24 h) as previously described [24]. Briefly, newborn rats were anesthetized with isoflurane and decapitated. Spinal cord tissues were manually freed of meninges under a stereoscopic microscope, cut into small pieces (approximately 1 mm^3^), and then separated into cells by repeated pipetting using a 5-mL pipette until no macroscopic tissue was observed. Next, the suspension of NSCs was filtered through a 0.22-µm filter and centrifuged at 800×*g* for 6 min at RT. Finally, 5 × 10^5^ cells in suspension were transferred into a T25 culture flask and cultured at 37 °C with 5% CO_2_ (Thermo Fisher Scientific) for 7 days. NSC purity was confirmed by staining for Nestin. After 7 days in differentiation conditions, GFAP and Olig2 immunocytochemistry was performed to evaluate the multi-directional differentiation ability of NSCs [25]. Cells in the third to fifth passage were used for experiments.

### NSC differentiation

We first detect whether protein Apelin expressed in NSCs by immunofluorescence, then, to evaluate the effect of Apelin and its inhibitor ML221 on NSC differentiation, NSCs were cultured on poly-l-lysine (PL)-coated dishes with different dosages of Apelin or its inhibitor ML221. Subsequently, immunocytochemistry using antibodies against GFAP (astrocyte marker) and NF200 (neuron marker) was performed to assess NSC differentiation. In addition, mRNA levels of these markers were examined using specific primers and qRT-PCR, as described above.

### Cell counting kit-8 (CCK8) assay

CCK8 assay was performed with a CCK8 kit (Beyotime) according to the manufacturer’s protocol. Briefly, 2 × 10^4^ NSCs in 100 µL of induction medium were seeded in PL-coated 96-well plates, to which different dosages of Apelin or ML221 were added. After culture for 24 h, 48 h, or 14 d, CCK8 detection was performed by adding 10 µL of CCK8 reagent to each well for 1 h before analysis. Cell proliferation rates were calculated as the absorbance of treated cells compared with an untreated control group.

### Basso–Beattie–Bresnahan (BBB) scores

BBB scores [26] were used to evaluate rat hindlimb motor function at 1, 3, 7, and 14 dpi by two researchers who did not otherwise participate in experiments.

### Statistical analyses

Quantification was performed by researchers blinded to the present study, and experiments were independently conducted three times with at least six replicates for each group. The resulting data are expressed as the mean ± standard deviation (SD), as calculated by Prism 8.0.1 (GraphPad Software, San Diego, CA, USA). Student’s *t*-test was used to compare two groups. For multiple comparisons, data were analyzed by one-way ANOVA followed by Tukey’s post hoc test. Assessment of BBB scores was analyzed by two-way RM ANOVA. *P* < 0.05 was considered statistically significant.

## Results

### Apelin expression was decreased after SCI

**Fig. 1:**
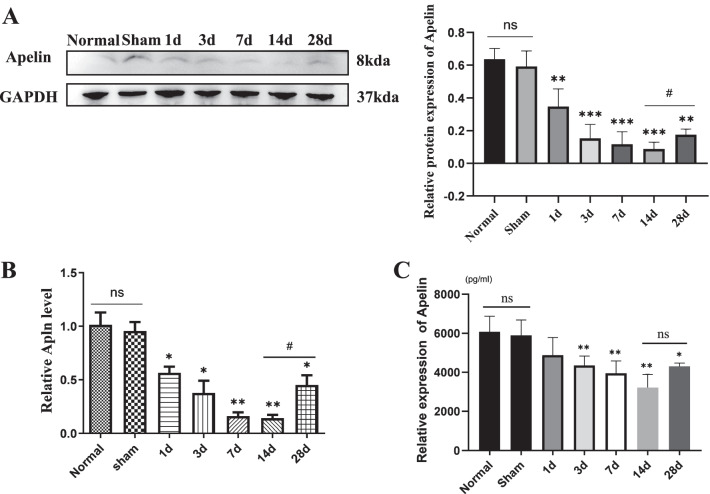
Expression of Apelin decreased after spinal cord injury (SCI). **A** Spinal cord sections obtained from healthy uninjured rats (control) and SCI rats on days 1, 3, 7, 14 and 28 after SCI; Apelin protein levels were normalized to that of GAPDH after western blotting. **B** Gene expression of Apelin was quantified in total RNA isolated from spinal cord tissue using qRT-PCR with specific primers; GAPDH was used as a loading control for qRT-PCR. **C** Serum Apelin was assayed by ELISA after SCI. (*p < 0.05, **p < 0.01 vs. Sham group, ^#^*p* < 0.05 14 d vs. 28 d, mean ± SD)

To evaluate Apelin expression after SCI, spinal cord tissues and serum from rats in normal, sham, and SCI groups were collected at 1, 3, 7, 14 and 28 days. Western blotting and RT-PCR results indicated that both protein and mRNA levels of Apelin decreased from 1 to 14 dpi (lowest level at 14 dpi) compared with sham groups, then increased at 28 dpi (*P* < 0.05, Fig. 1A, B), there was no significant difference in Apelin expression between normal and sham group (*P* > 0.05, Fig. 1A, B). Results of ELISA to quantify Apelin contents in peripheral blood showed the same results (Fig. 1C). Collectively, these results suggest that Apelin might be a candidate molecule related to SCI.

### Cellular distribution of Apelin after SCI

**Fig. 2:**
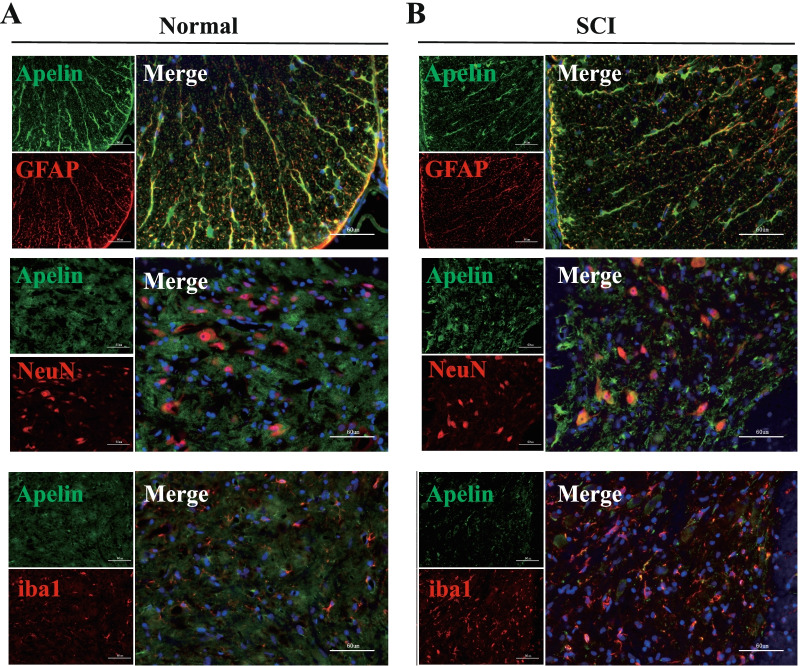
Changes of Apelin expression in spinal cord tissues during spinal cord injury. Immunofluorescence imaging showing co-localization of Apelin (green) with markers of specific cells types including NeuN (red, neurons), GFAP (red, astrocytes), and iba1 (red, microglia) in **A** normal spinal cord group and **B** 14 days after SCI; nuclei were counterstained with DAPI (blue). Co-localization appears yellow in the merged image. Scale bar = 60 µm

To investigate which cell types express Apelin in spinal cord tissue, double-immunofluorescence staining was performed after SCI; co-localization was indicated as a yellow fluorescent signal. As shown in Fig. 2, Apelin was expressed in GFAP (astrocytes), NeuN (neurons) and iba1 (microglia) positive cells, and the expression of Apelin was decreased after SCI (Fig. 2B) compared to the normal group (Fig. 2A).

### Characterizations of NSCs

**Fig. 3:**
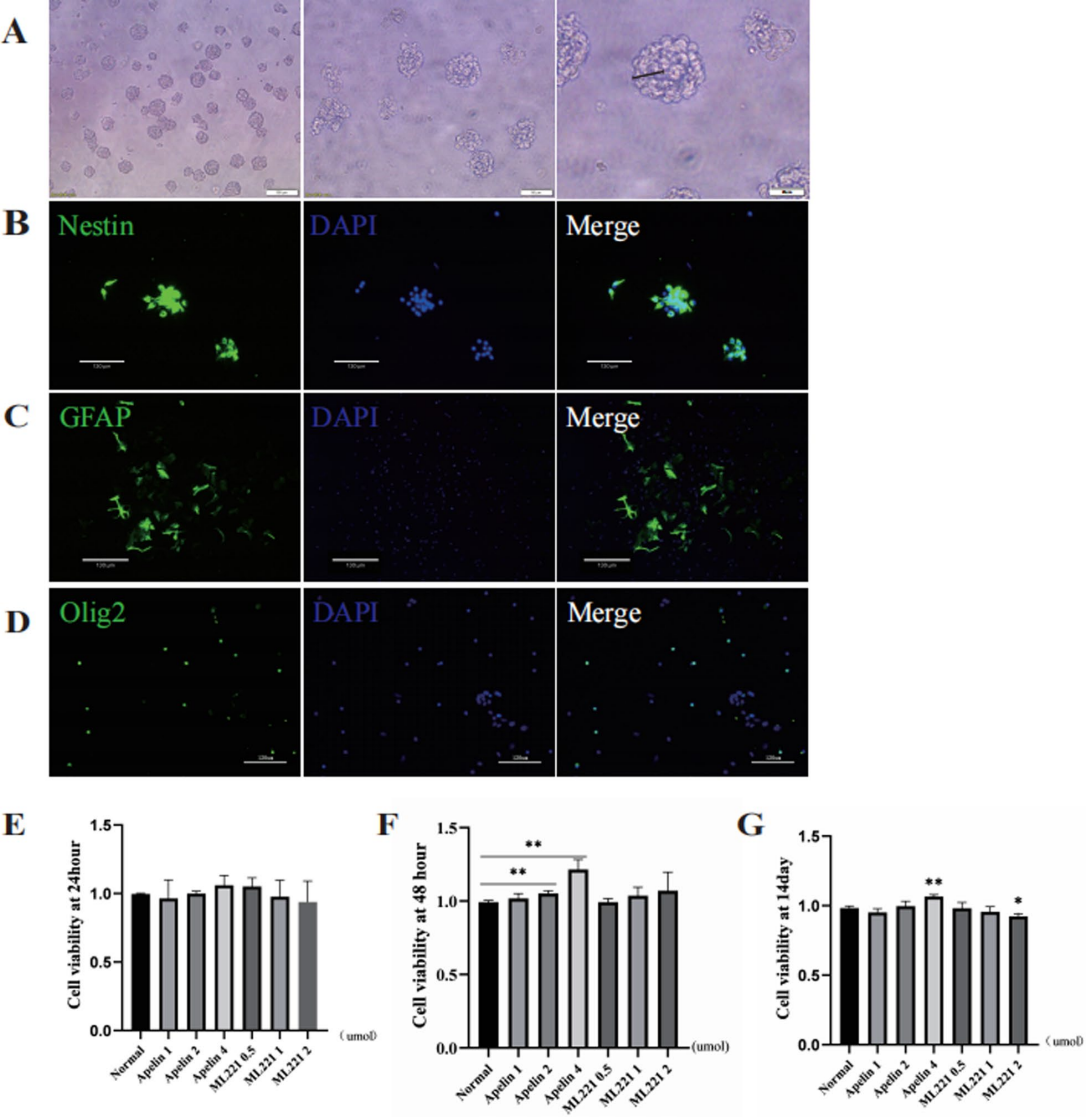
Identification of primary cultured neural stem cells (NSCs) and evaluation of their proliferation after administration of Apelin or its inhibitor ML221. **A** Morphology of NSCs under a light microscope, scale bar = 100 µm (left), 50 µm (middle) or 25 µm (right). **B** Identification of primary cultured NSCs, scale bar = 130 µm. **C**, **D** Multi-differentiation potential of NSCs was assessed by GFAP+ cells (green) and Olig2+ cells after 14 d, scale bar = 130 µm or 120 µm. Proliferation of NSCs under different dosages of Apelin and its inhibitor ML221 were determined by CCK8 assay at 24 h (**E**), 48 h (**F**), and 14 d (**G**) after SCI (*p < 0.05, **p < 0.01 vs. normal group, mean ± SD)

To characterize NSCs extracted from newborn rats, spherical neurospheres cultured in suspension were evaluated with fine light refraction under a light microscope (Fig. 3A). After seeding in PL-coated plates at 37 °C for 4 h, cells exhibited radial growth around the neurospheres. NSCs marker were employed to identify NSCs (Fig. 3B). Next, we evaluated the differential potency of NSCs by fluorescent staining of cells with GFAP (astrocyte) and Olig2 (oligodendrocyte). To evaluate differentiation potential, differentiated astrocytes and oligodendrocytes were identified (Fig. 3C, D). NSCs from third to fifth generations were collected for subsequent experiments.

### Apelin treatment promoted NSC proliferation

To investigate whether Apelin and its inhibitor ML221 could affect NSC proliferation, we conducted CCK8 assays. Third-passage NSCs were seeded into 96-well plates, and proliferation was evaluated 24 h, 48 h, and 14 d after the addition of each drug. As shown in Fig. 3E, after 24 h, no significant proliferation was detected. However, CCK8 assay results after 48 h (*P* < 0.05, Fig. 3F) indicated that cellular proliferation was significantly promoted by 2 µmol and 4 µmol Apelin, whereas ML221 administration did not elicit a statistically significant effect. After 14 d (Fig. 3G), 4 µmol Apelin promoted NSC proliferation while 2 µmol ML221 inhibited proliferation. Thus, 4 µmol Apelin and 2 µmol ML221 were selected as optimal dosages for subsequent experiments.

### Apelin and its inhibitor ML221 altered NSC differentiation

**Fig. 4:**
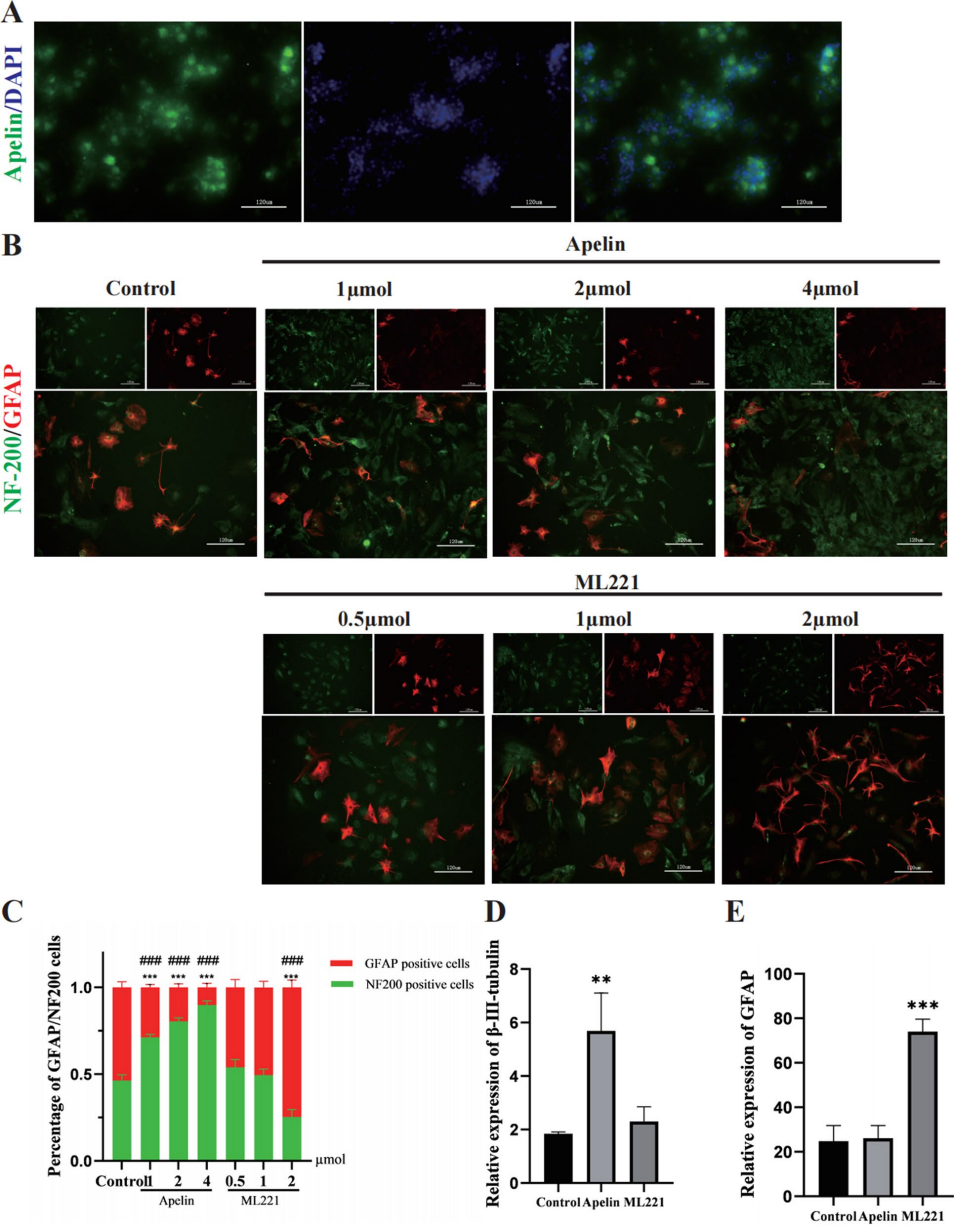
Apelin and ML221 promoted differentiation of NSCs. **A** Expression of Apelin (green) in NSCs (scale bar = 120 µm). **B** Differentiated cell types were identified by expression of specific markers via immunofluorescence of GFAP (astrocytes, red) and NF200 (neurons, green) under different dose of Apelin and ML221 (scale bar = 120 µm). **C** A statistic summary of NeuN+ and GFAP+ cells proportions (***p < 0.001 NF200 vs. control group, ^###^*p* < 0.001 GFAP vs. control group, mean ± SD). **D**, **E** RT-PCR results indicating different types of cells differentiated from NSCs following induction by Apelin and ML221. Primers for two targets were used: GFAP (astrocytes) and β-III-tubulin (neurons); a specific primer for GAPDH was also included in reactions (**p < 0.01, ***p < 0.001 vs. control group, mean ± SD)

Molecule Apelin were expressed in NSCs, to evaluate NSC differentiation after administration of Apelin or its inhibitor ML221, various dosages of Apelin and ML221 were added to the differentiation medium. As shown in Fig. 4B, after 14 d administration, Apelin promoted differentiation of NSCs into neurons (NF200, green) and reduced differentiation into astrocytes (GFAP, red), the proportion of neurons and astrocytes is statistically shown in Fig. 4C. To quantitatively analyze differentiation, qRT-PCR was conducted. The results indicated that Apelin promoted NSC differentiation into neurons (β-III-tubulin; *P* < 0.05, Fig. 4D) and had no significant effect on astrocytes (GFAP, Fig. 4D). Conversely, ML221 administration promoted differentiation of NSCs into astrocytes (GFAP; *P* < 0.05, Fig. 4E).

### iPSC transplantation promoted morphological and functional recovery after SCI

**Fig. 5:**
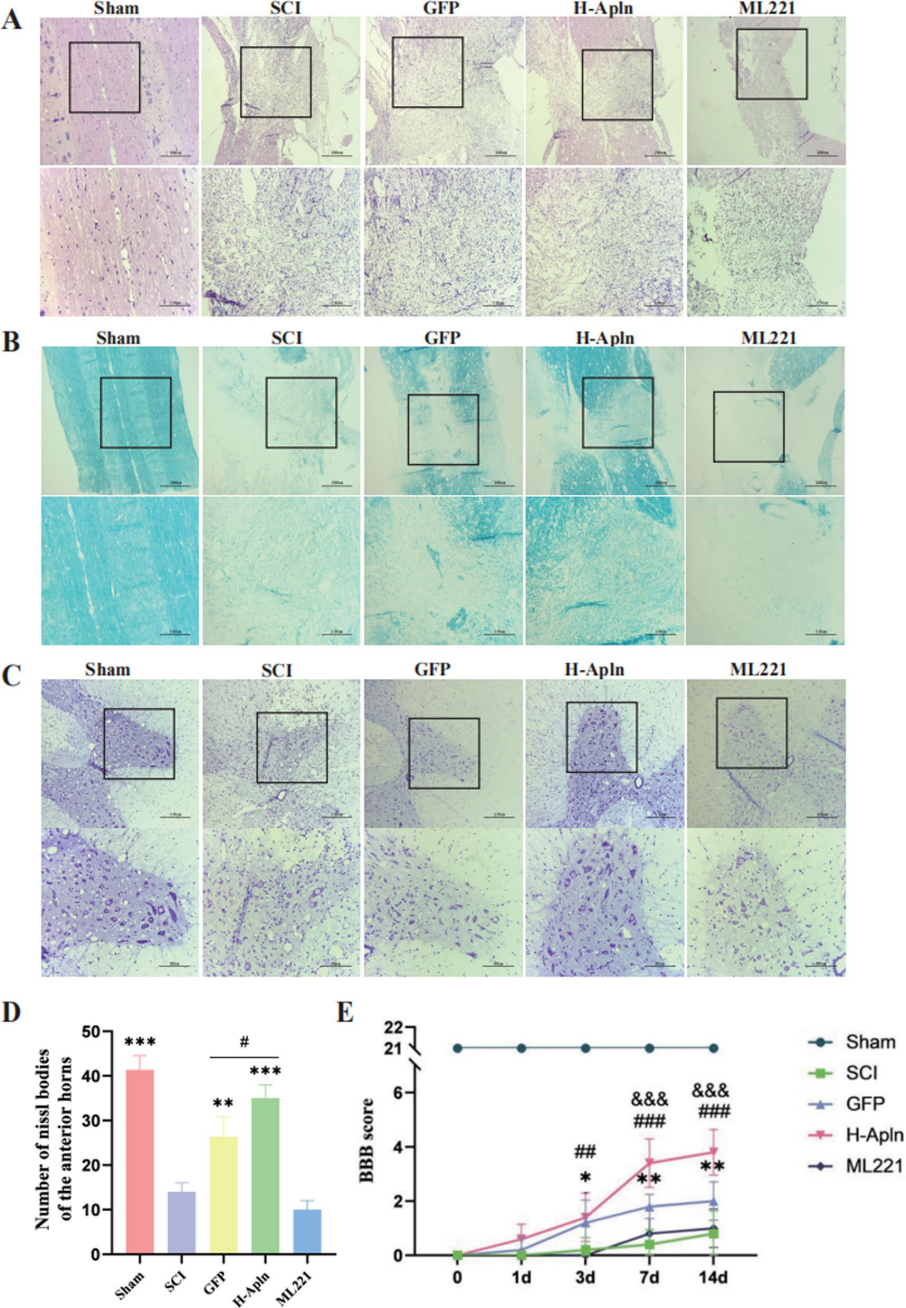
iPSC transplantation promoted morphological and functional recovery after SCI. **A** HE staining of spinal cord tissue at 14 d after injury, scale bar = 200 µm (top) or 110 µm (lower). **B** LFB staining was performed to identify demyelination at 14 d, scale bar = 200 µm or 110 µm. **C**, **D** Nissl staining to assess the loss of Nissl bodies at 14 d, and statistical graph showing numbers of Nissl bodies in each group (**p < 0.01, ***p < 0.001 vs. SCI group, ^#^*p* < 0.05 GFP vs. H-Apln group, mean ± SD). **E** Neurological function assessed by BBB score from 0 to 14 d after SCI (*p < 0.05, **p < 0.01 SCI vs. GFP group; ^##^*p* < 0.01, ^###^*p* < 0.001 SCI vs. H-Apln group, ^&&&^*p* < 0.001 GFP vs. H-Apln group, mean ± SD)

To determine the neuroprotective effect of transplanting lentivirus-infected iPSCs after SCI, rats were killed at 14 d post-injury. Next, animals were killed and their spinal cord tissues were collected. Morphometric analysis of HE, LFB, and Nissl staining of paraffin-embedded spinal cord tissue was performed. As shown in Fig. 5A, syringomyelia formed in longitudinal sections post-SCI, but transplantation of iPSCs improved spinal cord tissue recovery after SCI in GFP and H-Apln groups.

Results of LFB staining to evaluate myelination (Fig. 5B) showed that demyelination and degeneration occurred after SCI. Moreover, the H-Apln group exhibited significantly increased formation of myelin sheaths during SCI. However, administration of ML221 yielded more LFB unstained areas compared with the SCI group, suggesting more severe demyelination.

Nissl bodies were used as an indicator of neuronal survival. Nissl staining results indicated significant neuronal loss after SCI in the spinal cord anterior horn (*P* < 0.05 vs. Sham group; Fig. 5C, D). After transplantation of iPSCs overexpressing Apelin, the number of Nissl bodies was significantly increased (*P* < 0.05, Fig. 5D), but no statistical difference was observed between SCI and ML221 groups.

We followed up with behavioral testing at 1, 3, 7 and 14 d. BBB scores (Fig. 5E) suggested that iPSC administration promoted the functional recovery of rat hindlimbs after 3 dpi compared with the SCI group. Indeed, BBB scores of rats transplanted with iPSCs overexpressing Apelin were higher than SCI rats and GFP rats, while rats administered ML221 exhibited no functional changes.

In addition, SCI led to morphological degeneration of the spinal cord, which might form the basis of motor degeneration. These changes were reversed by iPSC transplantation, especially in the H-Apln group, indicating that Apelin might be an attractive target for SCI.

### Transplantation of iPSCs regulated microglia polarization and alleviated neuroinflammation

**Fig. 6:**
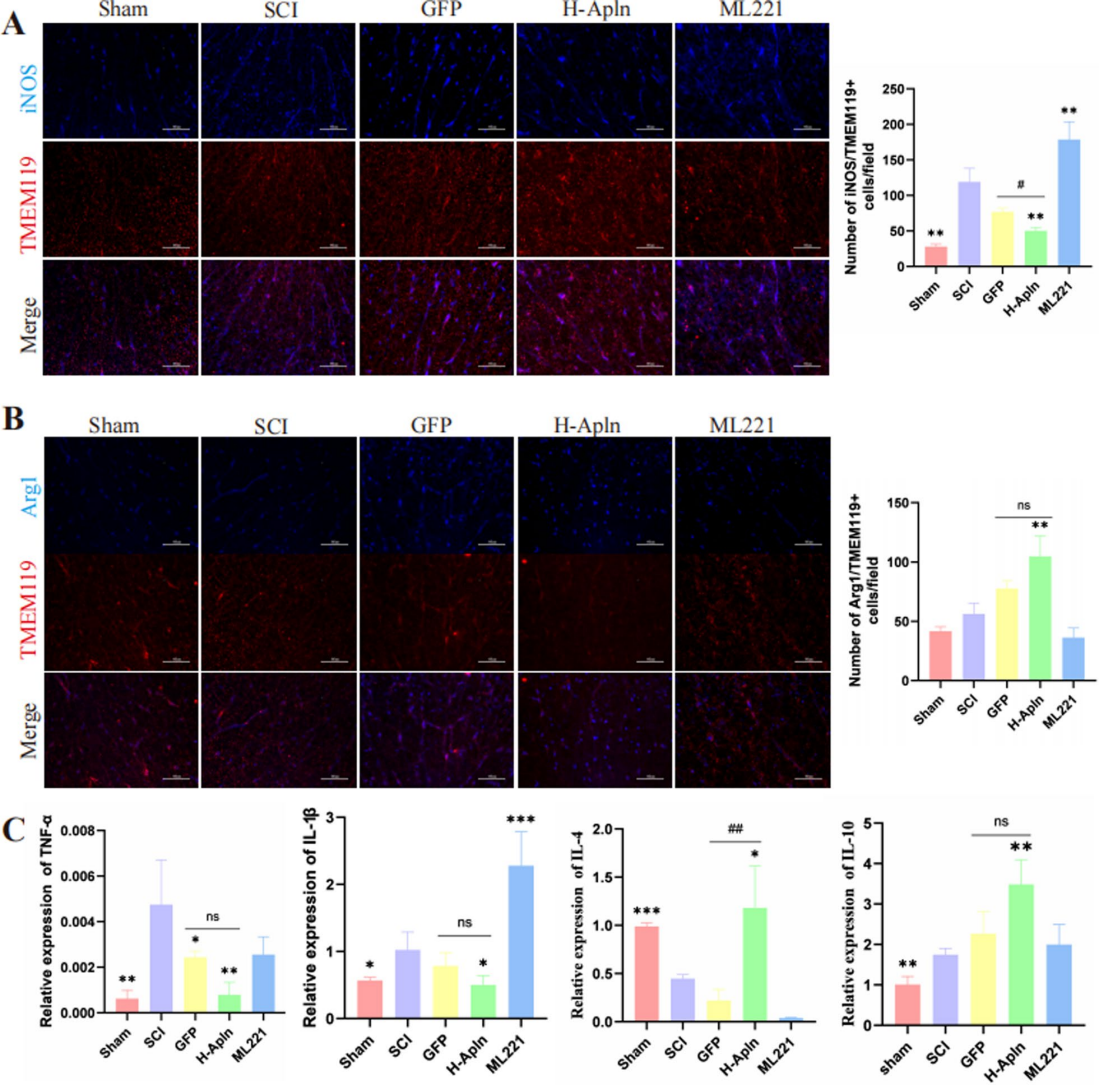
Transplantation of iPSCs regulated microglia polarization and alleviated neuroinflammation. **A**, **B** Microglia polarization were determined by immunofluorescence staining, TMEM119 was used to label microglia (red), and iNOS (blue) for M1 or Arg1 (blue) for M2 in samples collected at 14 dpi. after injury. Scale bar = 60 µm. **C** Relative expression of pro-inflammatory (IL-1β and TNF-α) and anti-inflammatory (IL-4 and IL-10) cytokines detected by qRT-PCR at 14 d post-injury (*p < 0.05, **p < 0.01, ***p < 0.001 vs. SCI group; ^##^*p* < 0.01 GFP vs. H-Apln, mean ± SD)

It was previously reported that microglia activation is the major source of neuroinflammation [27], M1 microglia are pro-inflammatory while M2 microglia are anti-inflammatory [5], therefore, we assessed M1/M2 polarization by immunofluorescence staining. M1 microglia were labeled with TMEM119+/iNOS+ and M2 microglia were labeled with TMEM119+/Arg1+. Immunofluorescence results indicated an increased number of M1 microglia in the SCI group compared with the sham group (*P* < 0.05, Fig. 6A), the number of M1 microglia were decreased in H-Apln group compared to the SCI group (*P* < 0.05, Fig. 6A). For TMEM119/Arg1-positive cells, the number of M2 cells were upregulated in H-Apln group compared to the SCI group (*P* < 0.05, Fig. 6B).

Next, we evaluated levels of pro- and anti-inflammatory factors by RT-PCR (Fig. 6C). RT-PCR results showed that transplantation of Apelin overexpressed iPSCs reversed the release of pro-inflammatory mediators and promoted release of anti-inflammatory factors (*P* < 0.05, Fig. 6C).

### Transplanted cells suppressed activation of A1 astrocytes

**Fig. 7:**
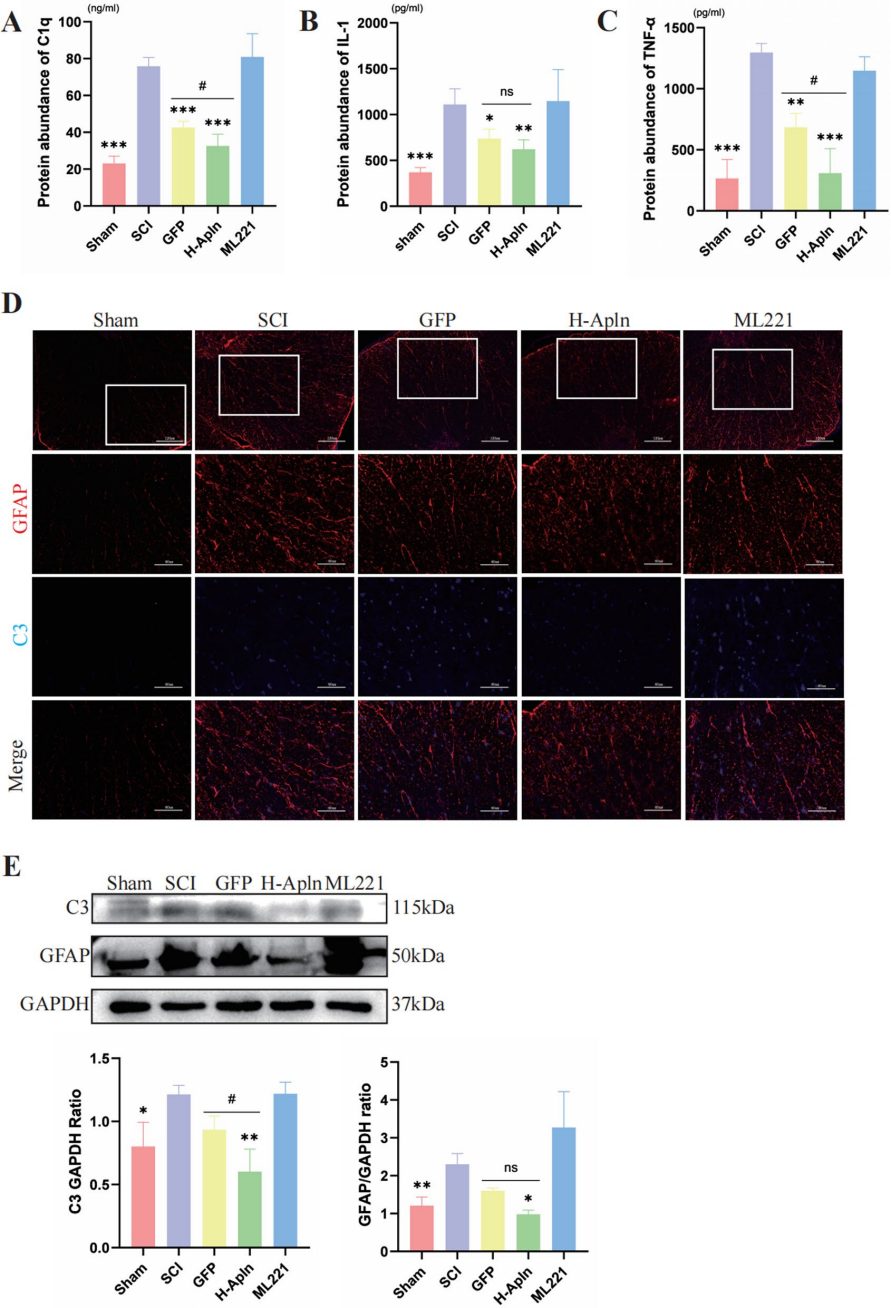
Cell transplantation reduced activation of A1 astrocytes. **A**–**C** Protein expression levels of pro-inflammation cytokines that promote A1 astrocyte activation were examined by ELISA post-SCI. (*p < 0.05, **p < 0.01, ***p < 0.001 vs. SCI group, ^#^*p* < 0.05, GFP vs. H-Apln, mean ± SD). **D** A1 astrocytes in spinal cord tissue double-labeled by GFAP (red) and C3 (blue); the sham group was used as a control, scale bar = 120 µm or 60 µm. **E** Western blotting of relative expression of GFAP and C3 in each group (*p < 0.05, **p < 0.01 vs.SCI group, ^#^*p* < 0.05, GFP vs. H-Apln, mean ± SD)

As described in the introduction, astrocytes can be divided A1 and A2 astrocytes [28]. A1 reactive astrocytes display neurotoxic properties and can induce neuronal degeneration [29]. In contrast, A2 astrocytes can elicit neuroprotective effects. In our study, we focused on A1 astrocytes to evaluate neuroinflammation. As previously reported, A1 astrocytes were activated by pro-inflammatory cytokines secreted by activated microglia, such as IL-1, TNF-α, and C1q [9]. Therefore, ELISA assays were used to detect levels of these cytokines in spinal cord tissue. As shown in Fig. 7A–C, very few pro-inflammatory cytokines were observed in the Sham group, but these cells were increased dramatically in the SCI group after 14 dpi (*P* < 0.05, Fig. 7A–C). In GFP and H-Apln groups, decreased release of pro-inflammatory factors was observed compared with the SCI group. Expression levels of IL-1 did not differ between these two groups, but expression of C1q and TNF-α was decreased in the H-Apln group compared with the GFP group (*P* < 0.05, Fig. 7C), indicating that transplanted iPSCs overexpressing Apelin yielded a better anti-inflammatory effect.

Complement component 3 (C3) is a characteristic marker of astrocytes [30]; accordingly, we identified A1 astrocytes using co-immunofluorescence staining for GFAP and C3. As shown in Fig. 7D, GFAP (red)/C3 (blue) double-positive cells were rarely seen in the Sham group, but rapidly increased in number in the SCI group. iPSC administration significantly decreased the level of C3, a phenomenon that was particularly evident in the H-Apln group. The results of western blotting to quantify protein expression of C3 and GFAP were consistent with immunofluorescence. In addition, administration of Apelin overexpressed iPSCs suppressed activation of A1 astrocytes, resulting in an anti-inflammatory effect.

### Transplantation of iPSCs overexpression Apelin promoted activation, proliferation, and differentiation of endogenous NSCs

**Fig. 8:**
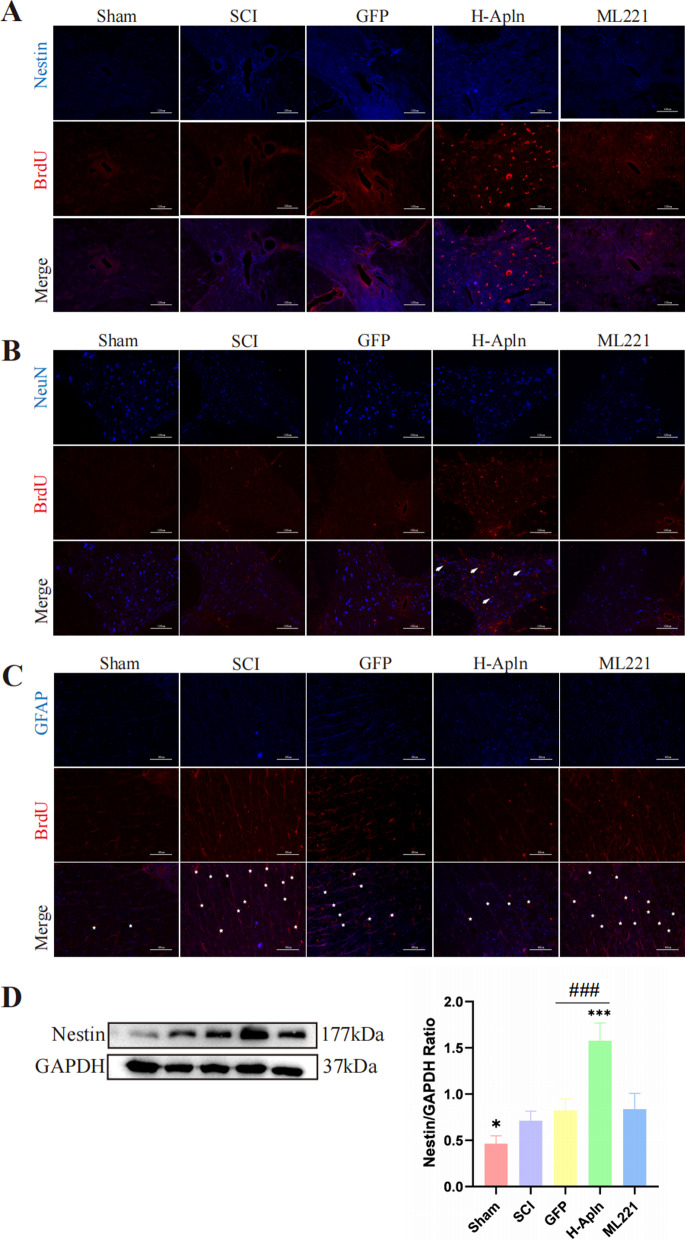
Activation and differentiation of endogenous neural stem cells after transplantation of iPSCs. **A** Activation and proliferation of endogenous NSCs was detected using BrdU (red)/Nestin (blue) co-immunofluorescence at 14 days after SCI in each group. **B**, **C** Double-immunofluorescence staining of NeuN (red) and BrdU to identify the differentiation of newly formed cells towards neurons (arrowheads), or GFAP (blue) and BrdU (red) to identify newly formed astrocytes (asterisk). **D** Western blotting analysis of Nestin protein expression (*p < 0.05 vs. SCI group, ^###^*p* < 0.001, GFP vs. H-Apln, mean ± SD)

Previous reports suggest that activation and migration of endogenous NSCs following SCI plays a key role in spontaneous self-repair [31, 32]. Therefore, we first used BrdU (50 mg/kg) incorporation to evaluate NSC proliferation. Immunofluorescence results revealed that transplantation of iPSCs increased the expression of Nestin/BrdU double-positive cells, suggesting that transplantation enhanced NSC activation and proliferation after SCI (Fig. 8A). Similar results were obtained with western blotting (Fig. 8D).

We next examined numbers of NeuN/BrdU double-positive cells (newborn neurons, Fig. 8B) and GFAP/BrdU double-positive cells (newborn astrocytes, Fig. 8C). Our results indicate that numbers of NeuN/BrdU double-positive cells were increased in the H-Apln group (arrowheads, Fig. 8B), however no differences were observed in the other groups. For GFAP/BrdU double-positive cells, numbers were increased significantly after SCI. Transplantation of iPSCs overexpression Apelin suppressed differentiation of NSCs toward astrocytes (asterisks, Fig. 8C). Among injured groups, the lowest number of astrocytes was observed in the H-Apln group.

Thus, we concluded that administration of iPSCs overexpressing Apelin can promote NSC differentiation into new neurons and suppress NSC differentiation towards astrocytes. Notably, this effect was most pronounced in the H-Apln group.

## Discussion

SCI, a severe CNS injury that can result in motor and sensory disorders [33], can be divided into primary and secondary injury [34]. SCI remain a challenging neurological disorder for which there is currently no cure. Therefore, it is of great importance to identify novel therapies.

Secondary injury is a main contributor that leads to long-term damage. Inflammatory responses contribute to neural injury phases and aggravate tissue injury. After CNS injury, astrocytes are activated by neuroinflammatory factors such as IL-1, TNF-α, and C1q secreted by microglia [28] in a process termed “astrogliosis” [7, 8]. As previously reported, A1 astrocytes can secrete neurotoxins that induce neuronal and oligodendrocyte cell death [28, 35, 36]. Moreover, astrogliosis leads to the formation of glial scars—a major inhibitor of axonal regeneration. Therefore, decreasing A1 astrocyte reactivity and secretion of pro-inflammation cytokines might be a promising strategy to alleviate functional loss after SCI.

Apelin, an endogenous peptide that binds the Apelin receptor APJ, can contribute to traumatic brain injury by suppressing autophagy [37–39], neuronal apoptosis, and inflammation. In addition, Apelin can reduce reactive oxygen species accumulation [37]; lower expression of pro-inflammation cytokines such as IL-1, IL-10, and TNF-α; increase Bcl2/Bax expression ratios; and reduce numbers of terminal deoxynucleotidyl transferase dUTP nick end labeling-positive cells. Another report suggests that intranasal delivery of Apelin-13 could promote angiogenesis and elicit neuroprotective effects after ischemic stroke in mice [40], indicating that Apelin can promote angiogenesis. Similar observations were made in the cardiovascular and reticuloendothelial systems [41]. Apelin has also been implicated in many neurological disorders such as Alzheimer’s, Parkinson’s, and Moyamoya diseases [42–46]. Taken together, Apelin/APJ has unique functions in regulating cell proliferation, apoptosis, pro-inflammatory activity, and revascularization. Therefore, we speculated that Apelin has similar functions in SCI.

Although several studies have investigated the effect of Apelin on neural system injury, we focused on its role in SCI. A previous study reported that Apelin could alleviate spinal cord ischemia/reperfusion injury by suppressing autophagy [47], while another found that intraperitoneal Apelin administration could suppress secretion of pro-inflammatory cytokines and promote behavioral recovery of rats with spinal cord contusion [48]. To verify the effect of Apelin, intracerebroventricular administration and intranasal delivery of Apelin-13 have also been used as delivery modes [40, 49]. However, because the half-life (t1/2) of Apelin was previously noted to be < 5 min [16], it is necessary to find a new delivery system. Previously, a novel liposomal nanocarrier system was used to deliver and prolong the duration of [Pyr1]-Apelin-13 as a therapeutic molecule into the injury site of a mouse model of transverse aortic constriction [50]. In our study, we attempted to use iPSCs as a carrier for drugs with a short half-life.

To clarify the effect of Apelin on SCI, we first detected spatiotemporal variations in Apelin expression after SCI. Our results show that Apelin expression decreased after SCI compared with normal and sham groups. The lowest expression was detected at 14 dpi, and subsequently Apelin was re-upregulated at 28 dpi. Next, we found that Apelin was located in neurons, astrocytes and microglia, suggesting it might be a potential involvement in SCI.

Stem cell therapy is considered an effective method for treating SCI [51]. However, some reports suggest that stem cells transplanted after SCI survive only 1–2 weeks. Accordingly, promoting proliferation and differentiation of endogenous NSCs might be a promising approach [52]. Hence, we extracted NSCs from rat spinal cord and treated them with different dosages of Apelin or its inhibitor ML221 to evaluate effects on NSC proliferation and differentiation. Our results show that 4 µmol Apelin-13 promoted NSCs differentiation into neurons and reduced NSCs differentiation into astrocytes. CCK8 assay results also indicated that Apelin could promote NSC proliferation. Collectively, the results of these in vitro experiments lay the groundwork for further in vivo studies.

To further detect the efficacy of Apelin in vivo, we intraspinally administered Apelin immediately after injury. However, because Apelin has a short half-life in the body, as low as 5 min [16], we used iPSCs as a carrier. Lentivirus-infected iPSCs overexpressing Apelin were transplanted into the injured spinal cord to observe treatment effects.

Our findings show that our administration alleviated the morphological and functional alterations induced by SCI. Moreover, HE staining indicated that this intervention augments spinal cord tissue repair. Nissl staining showed that transplantation of iPSCs overexpressing Apelin increased the number of Nissl bodies, indicating alleviation of neuronal injury. In addition, LFB staining suggested an increase in myelinated axons following iPSC transplantation. In sum, our study indicated that iPSCs overexpressing Apelin could alleviate morphological damage and improve motor function in the hindlimbs (BBB scores) of rats.

After SCI, excessive activation microglia and astrocytes can be induced [53]. Spinal cord injury can prime microglia toward the M1 phenotype, which is responsible for provoking neuroinflammation. In our results, iPSCs overexpressing Apelin administration alleviated the activation of M1 and promoted the polarization of M2 microglia, meanwhile, iPSCs administration regulated the inflammatory cytokines, iPSCs overexpressing Apelin were achieved better results. Then we detected the inflammatory cytokines by RT-PCR, our results indicated that transplantation of Apelin overexpressed iPSCs downregulated pro-inflammatory cytokines and upregulated anti-inflammation cytokines. In sum, transplantation of iPSCs overexpressing Apelin decreased microglial activation and regulated inflammatory cytokines in injured tissue, thus limiting tissue damage and pro-inflammatory reactions.

As previously reported, astrocytes play multifaceted roles after SCI [54]. On one hand, the response of astrocytes is important for restricting neuroinflammation and restoring tissue integrity during the acute phase of SCI [55]. On the other hand, excessive activation of astrocytes is related to glial scar formation, which is thought to prevent axonal regeneration [56]. However, recent studies indicate that astrocytes can be divided into two phenotypes: A1 neurotoxic astrocytes and A2 neuroprotective astrocytes [57]. A1 astrocytes are activated by pro-inflammation cytokines including IL-1, TNF-α, and C1q, which are secreted by microglia [58]; thus, we used ELISA to detect levels of these cytokines. Our results indicate that these pro-inflammatory factors were significantly decreased in GFP and H-Apln groups; moreover, TNF-α was lower in the GFP group compared with the H-Apln group. Recent studies reported that C3 is expressed on A1, but not A2, astrocytes; therefore, we determined numbers of A1 astrocytes by C3/GFAP double-positive immunofluorescence [59]. Our results show that numbers of C3/GFAP double-positive cells in the H-Apln group were lower compared with other injured groups; western blotting results further supported these results. In addition, transplantation of iPSCs overexpressing Apelin reduced the secretion of pro-inflammation factors and numbers of A1 reactive astrocytes, thereby alleviating the inflammatory response.

Although NSC activation is considered to play an important role in spontaneous self-repair [60], SCI provides a hostile microenvironment for transplanted cells with regard to their survival. Thus, inducing endogenous NSC proliferation and differentiation is a promising strategy. We therefore examined whether administration of Apelin could promote endogenous NSC proliferation using BrdU-incorporation experiments. BrdU+/Nestin+ cells represented activated endogenous NSCs after SCI. Our results show that transplantation of Apelin overexpressed iPSCs promoted activation and proliferation of endogenous NSCs after SCI, as well as repair of the injury.

We next identified newborn neurons and astrocytes by BrdU+/NeuN+ and BrdU+/GFAP+ immunofluorescence. Our results show that iPSCs overexpressing Apelin promoted the survival of newborn neurons, but this phenomenon was not seen in other groups. In addition, we observed large number of newborn astrocytes in the injured groups, with the exception of the H-Apln group, similar to the results of in vitro experiments.

## Conclusion

Altogether, our study demonstrates that Apelin expression was decreased after SCI in both neurons, astrocytes and microglia, indicating a potential role in SCI process. To evaluate this hypothesis, we used iPSCs as a carrier to transplant Apelin into the injured spinal cord tissue of rats, and observed the treatment effect. Our results show that administration of iPSCs overexpressing Apelin repaired the disrupted architecture caused by SCI; improved motor function in the hindlimbs; regulated microglial and astrocyte polarization; regulated the expression of inflammatory cytokines; promoted the activation, proliferation, and differentiation of NSCs (both in vivo and in vitro); and, finally, resulted in functional recovery after SCI.

## Data Availability

All data generated during and/or analysis during the current study are included in this published article.

## Abbreviations

ANOVA: Analysis of variance
NEC: Central nervous system
NSCs: Endogenous neural stem cells
iPSCs: Induced pluripotent stem cells
GFAP: Glial fibrillary acidic protein
IL: Interleukin
SCI: Spinal cord injury
PBS: Phosphate buffered saline
ELISA: Enzyme-linked immunosorbent assay
TNF-α: Tumor necrosis factor alpha
HE: Hematoxylin–eosin
BBB: Basso, Beattie, and Bresnahan
